# Oxidative stress is increased in critically ill patients according to antioxidant vitamins intake, independent of severity: a cohort study

**DOI:** 10.1186/cc5068

**Published:** 2006-10-13

**Authors:** Jimena Abilés, Antonio Pérez de la Cruz, José Castaño, Manuel Rodríguez-Elvira, Eduardo Aguayo, Rosario Moreno-Torres, Juan Llopis, Pilar Aranda, Sandro Argüelles, Antonio Ayala, Alberto Machado de la Quintana, Elena Maria Planells

**Affiliations:** 1 Nutrition and Dietary Unit, Virgen de las Nieves Hospital, Fuerzas Armadas Avenue, 18014 Granada, Spain; 2 Critical Care Unit, Virgen de las Nieves Hospital, Fuerzas Armadas Avenue, 18014 Granada, Spain; 3 Institute of Nutrition, Physiology Department, University of Granada, Campus de la Cartuja, 18071 Granada, Spain; 4 Biochemistry Department, University of Seville, Profesor García Gonzales street, 41012 Seville, Spain

## Abstract

**Introduction:**

Critically ill patients suffer from oxidative stress caused by reactive oxygen species (ROS) and reactive nitrogen species (RNS). Although ROS/RNS are constantly produced under normal circumstances, critical illness can drastically increase their production. These patients have reduced plasma and intracellular levels of antioxidants and free electron scavengers or cofactors, and decreased activity of the enzymatic system involved in ROS detoxification. The pro-oxidant/antioxidant balance is of functional relevance during critical illness because it is involved in the pathogenesis of multiple organ failure. In this study the objective was to evaluate the relation between oxidative stress in critically ill patients and antioxidant vitamin intake and severity of illness.

**Methods:**

Spectrophotometry was used to measure in plasma the total antioxidant capacity and levels of lipid peroxide, carbonyl group, total protein, bilirubin and uric acid at two time points: at intensive care unit (ICU) admission and on day seven. Daily diet records were kept and compliance with recommended dietary allowance (RDA) of antioxidant vitamins (A, C and E) was assessed.

**Results:**

Between admission and day seven in the ICU, significant increases in lipid peroxide and carbonyl group were associated with decreased antioxidant capacity and greater deterioration in Sequential Organ Failure Assessment score. There was significantly greater worsening in oxidative stress parameters in patients who received antioxidant vitamins at below 66% of RDA than in those who received antioxidant vitamins at above 66% of RDA. An antioxidant vitamin intake from 66% to 100% of RDA reduced the risk for worsening oxidative stress by 94% (ods ratio 0.06, 95% confidence interval 0.010 to 0.39), regardless of change in severity of illness (Sequential Organ Failure Assessment score).

**Conclusion:**

The critical condition of patients admitted to the ICU is associated with worsening oxidative stress. Intake of antioxidant vitamins below 66% of RDA and alteration in endogenous levels of substances with antioxidant capacity are related to redox imbalance in critical ill patients. Therefore, intake of antioxidant vitamins should be carefully monitored so that it is as close as possible to RDA.

## Introduction

Critically ill patients suffer from oxidative stress caused by reactive oxygen species (ROS) and reactive nitrogen species (RNS) [1,2]. Although ROS/RNS are constantly produced under normal circumstances, critical illness can drastically increase their production. Sources of oxidative stress during critical illness include activation of phagocytic cells of the immune system, production of nitric oxide by the vascular endothelium, release of iron and copper ions and metalloproteins, and ischaemia/reperfusion-induced tissue damage. ROS/RNS have also been reported to trigger the release of cytokines from immune cells, activate inflammatory cascades and increase the expression of adhesion molecules [3]. Inflammation and tissue injury result in an accumulation of granulocytes in organs, leading to greater generation of ROS, which further perpetuates or increases the inflammatory response and consequent tissue injury [4].

Critically ill patients have reduced plasma and intracellular levels of antioxidants and free electron scavengers or cofactors, and decreased activity of the enzymatic system that is involved in ROS detoxification [5]. The pro-oxidant/antioxidant balance is of functional relevance during critical illness because it is involved in the pathogenesis of multiple organ failure [6-9]. Moreover, the antioxidant capacity (AOC) of patients with sepsis may be compromised by increased utilization of plasma-binding proteins as part of the acute inflammatory response and by inadequate nutrition [8].

Recent clinical studies reported on the effects of prophylactic administration of antioxidants, as a component of nutritional support or as an individualized intervention, to patients at risk for oxidant-related complications [10]. Increased free radical generation and damage in critically ill patients has been associated with greater morbidity and mortality [11]. Against this background, we undertook the present study of various oxidative stress parameters in the serum of patients at admission to an intensive care unit (ICU) and on ICU day 7. The aim of the study was to identify any associations between oxidative stress in critically ill patients and their antioxidant vitamin intake and severity of illness. Three generic biomarkers of oxidation were measured: lipid peroxides (LP) and carbonyl groups (CO; which represent damage to lipids and proteins, respectively), and AOC of serum (which provides a measure of the overall protection against oxidative damage) [11]. Furthermore, assessment of oxidative stress should enable selection of an optimal formula for exogenous supply of antioxidant and protective compounds to re-establish redox balance in critical illness.

## Materials and methods

The study was conducted in consecutive patients admitted to the ICU of the Virgen de las Nieves Hospital (Granada, Spain) who met the following criteria: age 18 years or greater, clinical situation preventing oral nutrition, and expected ICU stay seven days or greater. The study was approved by the Clinical Research and Ethics Committee of our hospital.

Patients' demographic and clinical characteristics were recorded at study enrolment (ICU admission); these include age, sex, admission Acute Physiology and Chronic Health Evaluation (APACHE) II score and diagnosis. The Sequential Organ Failure Assessment (SOFA) score was calculated at ICU admission and at day seven. The difference in mean SOFA score between admission and day seven was calculated (SOFA change).

### Oxidative stress biomarkers

A number of assays may be used to measure the rates at which oxidative damage is occurring in living organisms and their ability to protect against such damage. These tests variously measure rates of excretion of damaged biomolecules in blood or urine. The markers used in the present study were chosen because of the simplicity of their measurement and because they still appear to yield useful information in the area of free radical research. Several markers were measured because the most accurate and clinically relevant approach to evaluating oxidative damage is to measure many different types of damage from different biomolecules. Blood samples were drawn for assessment of biomarkers immediately after stabilization of the patient and on day seven of ICU admission, using vacutainer tubes containing a solution of EDTA/K_3 _as anticoagulant. Samples were centrifuged at 2500 *g *for 15 minutes at 4°C. Plasma was separated and immediately frozen to -80°C until required for analysis (no later than 30 days).

LP are among the most important ROS generated by free radical action [12]. There are several different methods that may be used to detect them, which leads to a light emission or colour production. The method used in the present study [13] was chosen not only because of its simplicity but also because previous results from our group indicate that LP in serum may be useful in predicting oxidative stress in tissues [14]. This method measures the actual amount of lipid hydroperoxides (not a product of LP damage). Briefly, Fox reagent was prepared with 100 μmol/l orange xylenol, 4 mmol/l butylated hydroxytoluene, 25 mmol/l sulphuric acid and 250 μmol/l ammonium ferrous sulphate. Samples were mixed in vials with 900 μl Fox reagent and 35 μl methanol and then incubated at room temperature for 30 minutes. Vials were centrifuged at 2400 *g *for 10 minutes. Absorbance of the supernatant was measured at 560 nm (ε = 4.3 × 10^4^M^-1 ^cm^-1^).

CO are usually formed by oxidative mechanisms and reflect changes in proteins that have been exposed to oxidants. Several reports concerning oxidative stress have used this measurement to show the presence of the oxidative stress in proteins in human tissues [15-17]. CO content was determined by a spectrophotometric method using the reagent 2,4-DNPH (dinitrophenylhydrazine) [18]. A solution of 10 nmol/l 2,4-DNPH in 10% trifluoroacetic acid (15% final concentration) was added to the serum samples. The mixture was washed three times with 1 ml ethanol/ethyl acetate (1:1 vol:vol) to remove free residues of 2,4-DNPH. Samples were then re-suspended in 6 mol/l of guanidine in 50% formic acid overnight at room temperature. The CO content was determined by absorbance at 360 nm using a molar extinction coefficient of 22.000 M^-1 ^cm^-1^.

Total AOC is a parameter that provides information regarding the total charge of antioxidants present in the fluid under consideration. This index is frequently considered a useful indicator of a system's ability to regulate damage caused by ROS [19]. Among the methodologies used to evaluate total antioxidant activities, the most widely used colorimetric method for serum and plasma samples are ABTS (2,2'-azinobis-3-ethylbenzothiazoline-6-sulfonate)-based methods. AOC was determined using the ABTS method described by Villaño and coworkers [20] in 2005. Pre-oxidation of ABTS was performed in the presence of H_2_O_2 _and peroxidase. Once ABTS radical cation (ABTS•^+^) was formed, the reaction was started by adding an aliquot of the sample. Absorbance at 414 nm was measured. Standard Trolox solutions were also evaluated against the radical.

The endogenous antioxidants bilirubin, total proteins and uric acid were also measured in each patient using techniques validated in our setting.

### Nutrition

Nutritional support followed the protocol established by the Clinical Nutrition Unit of our hospital, based on American Society for Parenteral and Enteral Nutrition guidelines [21]. A daily nutritional log was kept for each patient (type, volume and composition of intake, tolerance, among other factors) from ICU admission for seven days.

The energy, macronutrients (carbohydrates, proteins and fats) and antioxidant vitamins (vitamins A, E and C) received (enterally or parenterally) by patients were calculated and compared with their recommended intake. The relation between vitamin intake and recommendation for each patient was calculated by using the recommended dietary allowance (RDA) adapted for enteral and parenteral nutrition [22].

### Statistical analysis

The main outcomes followed in the study were the continuous variables plasma protein CO, plasma LP and plasma total AOC. These results were used in the building of categorical variables, in which oxidative stress was considered to have worsened in each patient when two of the three parameters assessed worsened with respect to the initial assessment.

Antioxidant vitamin intake was expressed as percentage of RDA. Dummy variables for caloric intake (tertile II: 33% to 66% of RDA; tertile III: >66% of RDA) were employed. The patients were allocated to one of two cohorts according to vitamin intake; group 1 included patients whose intake of antioxidant vitamins was below 66% of RDA (tertile II), and group 2 included patients whose intake of these vitamins was between 66% and 100% of RDA (tertile III).

The distributions of variables between admission to the ICU and day seven and between groups were analyzed. First, the Kolmogorov-Smirnov test was applied to test the normal distribution of continuous variables (*P *> 0.05 for normality). For variables with a normal distribution, the comparison of absolute means between groups was performed using Student's *t *test, for continuous data that were not normally distributed the Wilcoxon test was used, and the percentage distribution of categorical variables was evaluated using the χ^2 ^test. Correlations between groups in quantitative variables were studied using Pearson's and Spearman's coefficient of correlation. In order to assess associations between adequate administration of antioxidant vitamins and oxidative stress, the relative risk was estimated.

Among the clinical and nutritional variables studied, the only parameter that differed significantly between groups was SOFA change, and this variable was entered into a multivariate regression analysis in order to test the independence of the association between oxidative stress and SOFA score.

All data are expressed as means, standard deviations and percentages. The statistical software package SPSS.12 for Windows (SPSS Inc., Chicago, IL, USA) was used for all analyses. Statistical significance was defined as a two-tailed *P *< 0.05 for all analyses.

## Results

Over a ten month period 40 patients (31 males and nine females) were included; they were all consecutively admitted to the ICU and were studied over at least a seven day period. Their mean age was 62 ± 15 years (range: 28–81 years). In terms of antioxidant vitamin intake, assessment of the patients' diets revealed that 10 received between 66% and 100% of RDA, whereas the rest (30 patients) received less than 66% of RDA. Table 1 shows the characteristics of the patients on admission. There were no differences between the two groups (patients with antioxidant vitamin intake between 66% and 100% of RDA and those with antioxidant vitamin intake <66% of RDA) in sex, diagnosis, endogenous antioxidants, APACHE II score or SOFA score.

Table 2 summarizes the main clinical and nutritional observations in the two groups over the study period. There was a significantly greater mean difference in SOFA score between admission and day seven in the group with antioxidant vitamin intake below 66% of RDA, whereas there was no difference between groups in days on mechanical ventilation, mortality, length at ICU stay or nutritional variables.

Changes in oxidative stress biomarkers in the 40 patients between admission and day seven in the ICU are shown in Table 3. Bivariate analysis of correlation of biomarkers revealed a weak positive association between LP and CO (r^2 ^= 0.350, *P *= 0.031), whereas there was a strong negative correlation between LP and AOC (r^2 ^= -0.660, *P *= 0.000). AOC was positively correlated with the endogenous antioxidants total proteins, bilirubin and uric acid (r^2 ^= 0.533, *P *= 0.015; r^2 ^= 0.355, *P *= 0.031; and r^2 ^= 0.388, *P *= 0.016, respectively).

**Figure 1:**
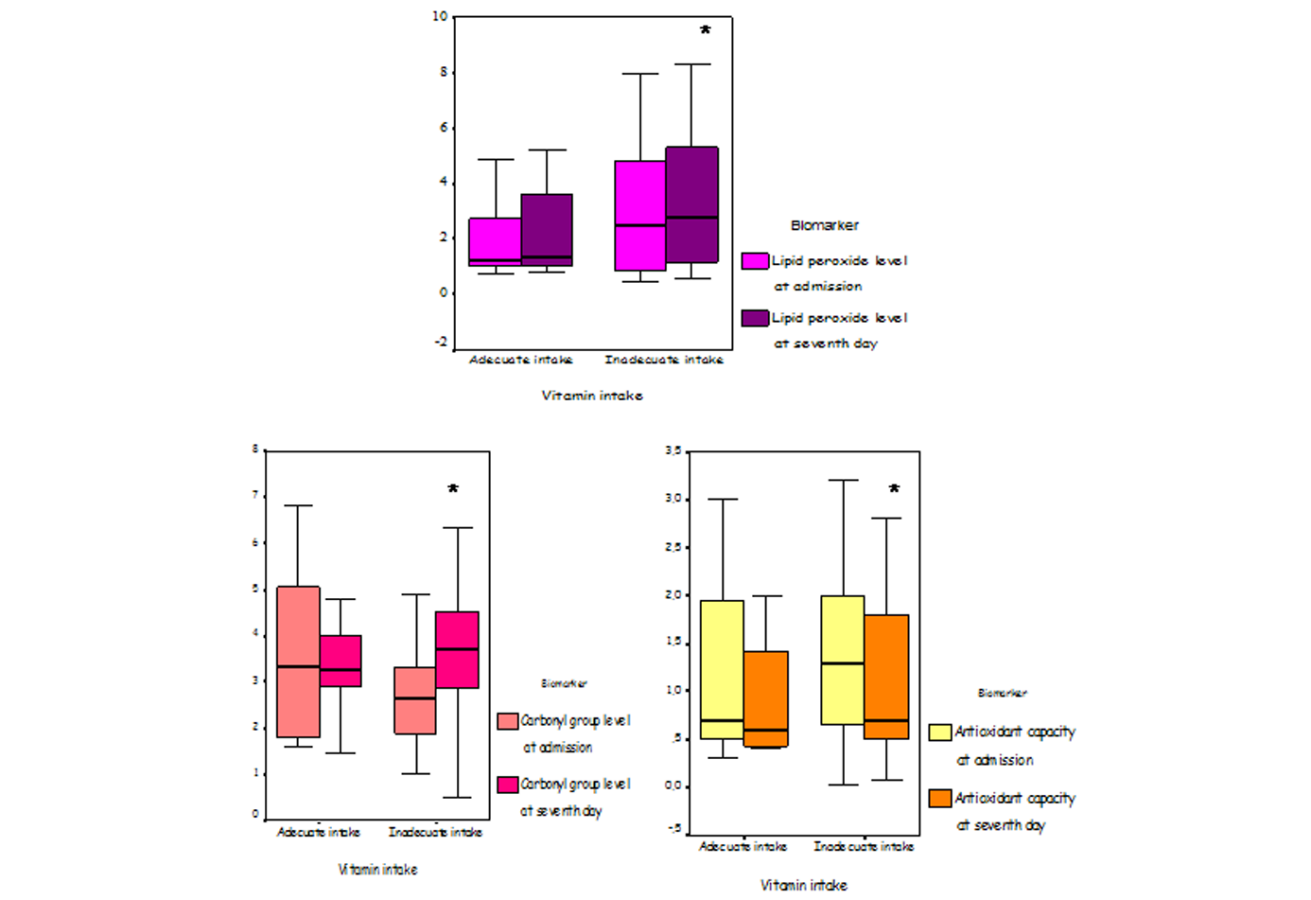
Changes in oxidative stress biomarkers according to vitamin intake.

Figure 1 shows the distribution of oxidative stress markers by study group (patients with intake of antioxidant vitamins <66% of RDA and those with intake of these vitamins between 66% and 100% of RDA). Patients with antioxidant vitamin intake below 66% of RDA had PL, CO and AOC levels of 2.4 nm/mg protein, 2.6 nm/mg protein and 1.5 nm/mg protein, respectively, at ICU admission; at day seven of the ICU stay these values were 2.9 nm/mg protein, 3.5 nm/mg protein and 1.1 nm/mg protein, respectively, and the change was significant in all cases (*P *< 0.01). However, no significant changes in these levels were observed in the group with better antioxidant vitamin intake.

**Figure 2:**
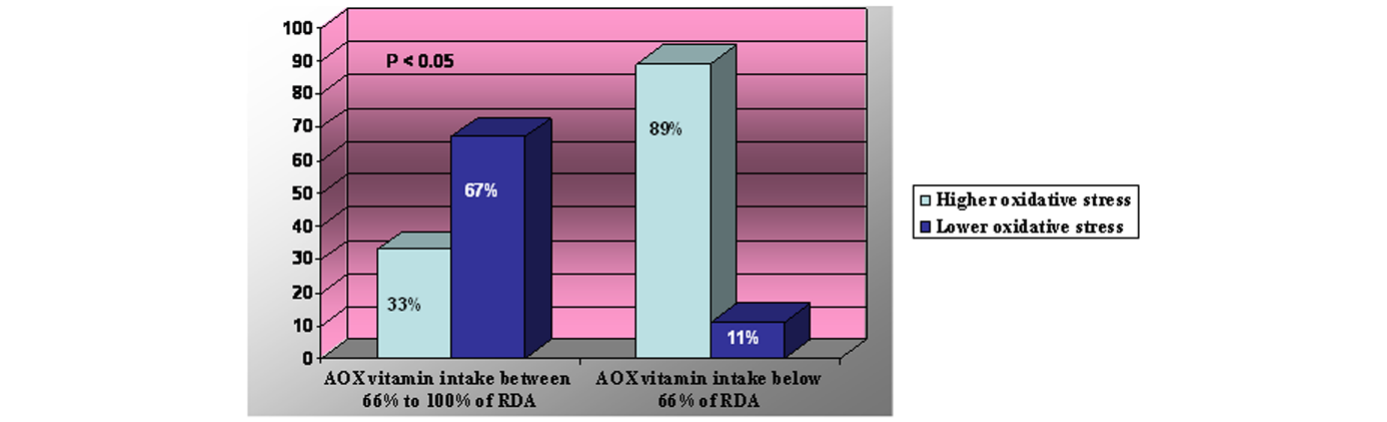
Worsening in oxidative stress according to intake of antioxidant vitamins. AOX, antioxidant; RDA, recommended dietary allowance.

There was a significantly greater (*P *= 0.003) worsening in oxidative stress parameters in those patients whose antioxidant vitamin intake was inadequate (<66% of RDA; Figure 2). We evaluated the risk for worsening oxidative stress as a function of antioxidant vitamin intake and found intake closer to the RDA to be protective (odds ratio [OR] = 0.06, 95% confidence interval [CI] = 0.010 to 0.39); this finding indicates that an intake of antioxidant vitamins from 66% to 100% of RDA reduced the risk for worsening oxidative stress by 94%.

In the bivariate analysis, patients with worsening oxidative stress exhibited a significant increase in SOFA score between ICU admission and day seven (from 6.7 to 8.27; *P *< 0.05), whereas patients with no change in oxidative stress markers exhibited no significant change in SOFA score. In the multivariate analysis, better intake of antioxidant vitamins (66% to 100% of RDA) emerged as an independent variable (OR = 0.079, 95% CI = 0.014 to 0.459), whereas SOFA change was no longer statistically significant (OR = 1.282, 95% CI = 0.81 to 2.01). Hence, the worsening in oxidative stress significantly differed between patients who received antioxidant vitamins from 66% to 100% of RDA and those who did not, regardless of changes in the severity of illness (SOFA score).

## Discussion

A major finding of the study was that administration of antioxidant vitamins at between 66% and 100% of RDA can reduce the risk for oxidative stress by 94%. Dietary antioxidants are known to play a fundamental role in protecting against ROS. Nathens and coworkers [23] concluded that early antioxidant supplementation with α-tocopherol and ascorbic acid in surgical patients reduces the incidence of organ failure and shortens the ICU length of stay. Presier and colleagues [24] compared critical care patients receiving dietary supplementation with antioxidant vitamins A, C and E versus control individuals, and they reported higher plasma concentrations of β-carotene, α-tocopherol and low-density lipoprotein, and greater resistance to low-density lipoprotein oxidation in the supplemented group. However, they found no difference in clinical outcomes between vitamin-treated patients and control individuals. Crimi and coworkers [25] observed that dietary enteral supplementation with vitamins C and E for 10 days prevented lipid peroxidation and oxidative stress in critical care patients and significantly influenced their clinical outcome at 28 days.

A relationship has been identified between oxidative stress and common critical care syndromes and diseases [8,26-28], with reports of an association between higher oxidative stress levels and more extensive organ dysfunction in ICU patients. However, no independent association was found in the present study between oxidative stress and changes in clinical severity of illness (as indicated by SOFA score) over the study period. According to the findings presented here, oxidative stress markers do not worsen in patients with antioxidant vitamin intake from 66% to 100% of RDA, even among those with greater deterioration in SOFA score. In contrast, other investigators found a significant relationship between oxidative stress and severity in critically ill patients. Cowley and colleagues [29] demonstrated a relation between onset of severe sepsis with secondary organ dysfunction and decreased plasma AOC in a population with a mean APACHE II score of 19.4, with a greater decrease in nonsurvivors than in survivors. Interestingly, those investigators found that survivors had responded to the insult with an increase in plasma AOC, which had remained persistently low in nonsurvivors. Goode and collegues and coworkers [8] observed increased free radical activity, measured as decreased antioxidant vitamin concentrations, increased lipid peroxidation and increased nitrite excretion, and found an association between plasma LP and organ failure in patients with septic shock (mean APACHE II score of 21). These discrepancies with the findings of the present study might be accounted for by higher mean APACHE II scores (worse prognosis) of the above series compared with the mean score of 15 in the present study.

Greater depletion of antioxidants has been related to a greater severity of trauma, systemic inflammatory response syndrome, or sepsis [30,31]. Low endogenous stores of antioxidants are associated with increased free radical generation, an augmentation in the systemic inflammatory response and consequent cell injury, increased morbidity and greater mortality in critically ill patients [32]. There is experimental and clinical evidence that critical care patients suffer from severe oxidative stress [33], and cellular redox imbalance is known to play a role in the pathogenesis of organ dysfunction in these patients. On the other hand, studies of haemodialyzed patients with sepsis revealed a paradoxically greater AOC in serum, which the authors attributed to the high levels of bilirubin and uric acid in the patients [34,35]. In the present study, the higher bilirubin concentration in patients with greater change in SOFA score may offer greater AOC, which may help to explain why these patients did not exhibit worsening oxidative stress despite their more severe clinical situation.

Various free radicals are produced as a consequence of normal cellular metabolism, and they are removed by endogenous scavengers under normal conditions in healthy tissues. However, the rate of release of reactive oxygen intermediates (ROI) is increased under pathological conditions [12], although their high reactivity and short life hampers their detection *in vivo*. Therefore, determination of oxidative damage markers (for example, LP and CO) and total AOC is of major value in assessing the effects of ROI in biological systems [11].

Oxygen reactive species react to all types of biological substrates and especially to polyunsaturated fatty acids. Reaction of ROI with these constituents of cellular membranes leads to lipid peroxidation, with increased plasma LP levels and cell membrane destruction [36]. Tsal and coworkers [37] recently observed that critically ill patients with systemic inflammatory response syndrome had significantly decreased AOC and increased lipid peroxidation in comparison with healthy control individuals, suggesting that patients with systemic inflammatory response syndrome have a disorder of redox balance.

In the present patients, a mean increase in plasma LP values of 24% was recorded from admission to day 7 of the ICU stay. Lipid peroxidation generates peroxyl radicals that, alone or with other compounds, can remove hydrogen from the fatty acid adjacent to the cell membrane, thereby extending the chain of lipoperoxidation reactions. Furthermore, products with a low molecular weight can escape from the membrane and produce disorders at more distant locations [38]. In an experimental study in rats, Argüelles and coworkers [14] found a statistically significant correlation between LP values measured in serum and those measured in tissues, indicating that serum LP levels may serve to predict oxidative status.

Proteins are also subject to oxidative damage by free radicals, leading to increased CO concentrations. Protein CO can be formed in various ways, not all of which depend on direct modification of the protein [8]. The determination of CO in biological compounds may not be appropriate for examining the effects of exposure of proteins to ROI. However, because CO is formed by oxidative mechanisms, their quantification may provide a reasonable index of oxidative stress status. The study conducted by Argüelles and coworkers [14] cited above revealed no correlation between CO in serum and that in different tissues, indicating that determination of the former only measures the damage produced in serum proteins. This may explain the low association found in the present series between CO and LP values, because LP would indicate oxidative damage in serum and tissues, whereas CO values only provide information on effects in serum.

Reduction in antioxidants is another component of the biological response to ROI, and a 20% reduction in serum AOC was observed in the present study. Different classes of antioxidants play a major role in the organism's defence system against the free radicals generated.

In the present study, a good correlation was found between the serum molecules that scavenge free radicals (total proteins, uric acid and bilirubin) and AOC. Of the AOC of plasma, 52% was accounted for by total proteins, 38% by uric acid and 1.5% by bilirubin. Erel [39] reported similar results, finding contributions to total AOC in serum of 52.9% by total proteins, 33.1% by uric acid and 2.4% by bilirubin.

Proteins are the main antioxidant component in serum, and their antioxidant effects are mainly produced by the free sulphydryl groups they contain. For many years bilirubin pigments were considered to be toxic products, formed during heme catabolism. However, recent studies have shown that bilirubin is a strong physiological antioxidant that can provide an important degree of protection against atherosclerosis, heart disease and inflammation [40]. Uric acid is another powerful antioxidant that captures and reduces the activity of free radicals [41].

## Conclusion

The critical condition of patients admitted to the ICU is associated with worsening oxidative stress. Intake of antioxidant vitamins below 66% of RDA and alteration in endogenous levels of substances with antioxidant capacity are related to redox imbalance in critically ill patients. Therefore, intake should be carefully monitored to ensure that the patient receives antioxidant vitamins as close as possible to the RDA.

## Key messages

• There was a significantly greater mean difference in SOFA score between admission and day seven in the group with antioxidant vitamin intake below 66% of RDA, whereas there was no difference between groups in days on mechanical ventilation or in nutritional variables.

• Patients with antioxidant vitamin intake below 66% of RDA had LP, CO and AOC levels of 2.4 nm/mg protein, 2.6 nm/mg protein and 1.5 nm/mg proteins, respectively, at ICU admission; these values were 2.9 nm/mg protein, 3.5 nm/mg protein and 1.1 nm/mg protein, respectively, at day seven of ICU stay, and this change was statistically significant in all cases (*P *< 0.01).

• There was a significantly greater (*P *= 0.003) worsening in oxidative stress parameters in patients who received lesser amounts of antioxidant vitamins.

• The worsening in oxidative stress significantly differed among patients who received antioxidant vitamins from 66% to 100% of RDA and those who did not, regardless of change in severity of illness (SOFA score).

## Abbreviations

ABTS = 2,2'-azinobis-3-ethylbenzothiazoline-6-sulfonate; AOC = antioxidant capacity; APACHE = Acute Physiology and Chronic Health Evaluation; CI = confidence interval; CO = carbonyl group; DNPH = dinitrophenylhydrazine; ICU = intensive care unit; LP = lipid peroxides; OR = odds ratio; RDA = recommended dietary allowance; RNS = reactive nitrogen species; ROI = reactive oxygen intermediates; ROS = reactive oxygen species; SOFA = Sequential Organ Failure Assessment.

## Competing interests

The authors declare that they have no competing interests.

## Authors' contributions

AJ carried out the oxidative stress assessment, participated in the sequence alignment and drafted the manuscript. RM, CJ and AE participated in the sequence alignment (patients chosen and clinical assessment conducted) and performed the statistical analysis. AS, AA and MA carried out the oxidative stress assessment. PA and MR participated in the design of the study. LJ, AP and PE conceived the study, participated in its design and coordination, and helped to draft the manuscript. All authors read and approved the final manuscript.

## Figures and Tables

**Table 1:** Baseline characteristics of the study population

Characteristic	Group 1 (antioxidant vitamin intake 66–100% of RDA; *n *= 10)	Group 2 (antioxidant vitamin intake <66% of RDA; *n *= 30)	*P*
Age (years; mean ± SD)	64 ± 14	53 ± 15	NS
Gender (male/female; *n *[%])	7 (18)/3 (7)	23 (58)/7 (17)	NS
Medical/surgical (*n*)	6/4	13/17	NS
Diagnosis (%)			NS
Respiratory			
ARDS	3	8	
Pneumonia	1	3	
Cardiovascular			
Postsurgical	0	4	
IHD	2	7	
Abdominal/hepatic disease			
Peritonitis	3	6	
Pancreatitis	1	2	
APACHE II score	13 ± 6.6	15 ± 4.1	NS
Admission SOFA score (mean ± SD)	6.6 ± 2.8	5.9 ± 2.5	NS
Bilirubin (mg/l)	1.6 ± 2.6	3.8 ± 2.5	NS
Uric acid (mg/l)	4.5 ± 2.6	3.7 ± 3.6	NS
Total proteins	5.0 ± 0.7	5.1 ± 0.740	NS

APACHE, Acute Physiology and Chronic Health Evaluation; ARDS, acute respiratory distress syndrome; IHD, ischaemic heart disease; NS, not significant; RDA, recommended dietary allowance; SD, standard deviation; SOFA, Sequential Organ Failure Assessment.

**Table 2:** Clinical and nutritional events recorded in the study

Events	Group 1 (antioxidant vitamin intake 66–100% of RDA; *n *= 10)	Group 2 (antioxidant vitamin intake <66% of RDA; *n *= 30)	*P*
Days on MV	4.0 ± 4.7	3.8 ± 2.2	NS
Mean SOFA change (mean ± SD)	0.77 ± 1	1.48 ± 2.14	0.035
Mortality (*n *[%])	4 (40)	18 (60)	NS
Length of ICU stay (days; mean ± SD)	35 ± 13	32 ± 11	NS
Type of nutrition (%)			NS
Parenteral	56 (5)	20 (6)	
Enteral	33 (3)	55 (17)	
Mixed (enteral and parenteral)	11 (2)	25 (7)	
Energy (mean ± SD)	1,299 ± 708	1,081 ± 495	NS
Nitrogen (mean ± SD)	11.82 ± 10.35	7.16 ± 5.41	NS
Carbohydrates (mean ± SD)	180 ± 91	182 ± 83	NS
Fats (mean ± SD)	47 ± 31	35 ± 27	NS

MV, mechanical ventilation; NS, not significant; RDA, recommended dietary allowance; SD, standard deviation; SOFA, Sequential Organ Failure Assessment.

**Table 3:** Oxidative stress biomarkers at ICU admission and day 7: patients with worsening oxidative stress versus those with no change in oxidative stress

Biomarkers	Worsening OS			Not worsening OS		
	
	At admission	Day 7	*P*	At admission	Day 7	*P*
LP (nm/mg protein)	2.14 ± 0.9	2.73 ± * 1.4	0.01	2.17 ± 0.9	2.13 ± 1.7	0.950
CO (nm/mg protein)	2.34 ± 1.0	3.49 ± * 1.2	0.00	3.54 ± 0.8	4.12 ± 1.4	0.216
AOC (nm/mg protein)	1.57 ± 0.8	1.15 ± * 0.8	0.02	1.13 ± 0.2	1.18 ± 0.8	0.673

Values are expressed as mean ± SD. *P < 0.05, versus admission value. AOC, antioxidant capacity; CO, carbonyl group; OS, oxidative stress; ICU, intensive care unit; LP, lipid peroxide; SD, standard deviation.
